# Getting rights right: implementing ‘Martha’s Rule’

**DOI:** 10.1136/jme-2023-109650

**Published:** 2024-01-06

**Authors:** Mackenzie Graham, Isabel Hanson, James Hart, Peter Young, Sapfo Lignou, Michael J Parker, Mark Sheehan

**Affiliations:** 1 Ethox Centre, Nuffield Department of Population Health, University of Oxford, Oxford, UK; 2 Nuffield Department of Primary Care Health Sciences, University of Oxford, Oxford, UK; 3 NIHR Oxford Biomedical Research Centre, Oxford, UK

**Keywords:** Ethics, Ethics- Medical, Medical Errors, Public Policy

## Abstract

The UK government has recently committed to adopting a new policy—dubbed ‘Martha’s Rule’—which has been characterised as providing patients the right to rapidly access a second clinical opinion in urgent or contested cases. Support for the rule emerged following the death of Martha Mills in 2021, after doctors failed to admit her to intensive care despite concerns raised by her parents. We argue that framing this issue in terms of patient rights is not productive, and should be avoided. Insofar as the ultimate goal of Martha’s Rule is the provision of a clinical service that protects patient safety, an approach that focuses on the obligations of the health system—rather than the individual rights of patients—will better serve this goal. We outline an alternative approach that situates rapid clinical review as part of a suite of services aimed at enhancing and protecting patient care. This approach would make greater progress towards addressing the difficult systemic issues that Martha’s Rule does not, while also better engaging with the constraints of clinical practice.

## Introduction

 The UK government has recently committed to adopting a new policy—dubbed ‘Martha’s Rule’—which would give patients a formal mechanism to rapidly access a second clinical opinion in urgent or contested cases. Support for the rule has emerged following a campaign by the parents of Martha Mills, who died of sepsis in 2021 after doctors failed to admit her to intensive care despite concerns raised by her parents.

The circumstances of this case reveal a complex set of problems with the way the health system functions and the way it can malfunction. These problems include examples of an unwillingness of healthcare practitioners (HCPs) to listen and properly reconsider a diagnosis and course of action, a lack of communication about or respect for the concerns of other HCPs or patients/carers, and the lack of formal mechanisms to circumvent these breakdowns in communication when they occur. Navigating these problems appropriately is not easy, but an ethical response to cases like Martha’s demands it.

While it is possible that if Martha had been reviewed by an independent clinical team she would have been moved to intensive care, the explanation for her death cannot be reduced simply to the lack of a right to obtain a second opinion. We argue that the subsequent response to Martha’s case—specifically, calls for the recognition of a right to rapidly obtain a second clinical opinion—does not adequately address the complex issues at work in this case and cases like it. Further, introducing such a right raises various ethical and practical problems of its own.

In what follows, we argue that framing this issue in terms of patient rights is not productive, and should be avoided. Insofar as the ultimate goal of Martha’s Rule is the provision of a clinical service that protects patient safety, an approach that focuses on the obligations of the health system—rather than the individual rights of patients—will better serve this goal. As an alternative to Martha’s Rule understood as a right to a second opinion, we argue for an approach that situates rapid clinical review as part of a suite of services aimed at enhancing and protecting patient care. This approach would make greater progress towards addressing the difficult systemic issues that Martha’s Rule does not, while also better engaging with the constraints of clinical practice.

## The ‘right’ to a second opinion

Martha’s Rule is modelled after other patient-activated rapid response (PARR) services currently in use both in the UK and internationally, whereby patients (or their carers) have a way to directly access an independent clinical team to request a review. However, there is some ambiguity about what Martha’s Rule could or might actually entail. Several news outlets,^1 2^ as well as a blog post by the think tank which drafted the original proposal for the rule,^3^ have characterised it as establishing a right to *obtain* a clinical review from an independent clinical team. However, other sources,^4^ including the original proposal,^5^ state that Martha’s Rule is the right to *request* a clinical review from an independent team.^1^ Whereas a right to request a rapid clinical review might be easier to satisfy in practice than a right to obtain one, the problem we see is in framing Martha’s Rule as a ‘right’ in the first place.

What does it mean to say that patients have the *right* to obtain, or even request, a second clinical opinion? The idea of a right is fundamentally connected to the idea of a duty or obligation. Roughly, if a person has a right to X, there is some other party (or parties) that has an obligation not to interfere with that person in securing X, or, depending on the nature of the right, to positively aid them in securing X. Thus, in specifying the nature of a right, we need to also specify the nature and content of the obligations that are conferred on others, as well as to whom these obligations apply.

The right of a patient to rapidly obtain a second opinion would thus obligate some other party to provide it—in this case, the health system and the HCPs working within it. What would such a right entail? Would a hospital be required to provide an independent review for any reason, in any circumstance? Would there be circumstances in which it would be appropriate to refuse a request for a second opinion (ie, in what ways would this right be ‘defeasible’)? What would be done in situations in which this right conflicted with another patient’s right (eg, two patients each seeking a rapid review from a single team, or if providing a second opinion necessitated a different patient not being seen in a timely manner)?

Specifying and incorporating a right to a rapid clinical review into the existing milieu of healthcare rights and obligations, while also accounting for the practical constraints of the current health system, would be an enormously complex task. It may be more productive to instead determine what we take the obligations of the health system to be, and work to devise a system that satisfies these obligations as favourably as possible. This will necessarily involve compromises of various kinds, as the practical constraints of the health system will make it impossible to satisfy every potential need or preference of every relevant stakeholder at all times. Insisting on something being a right can effectively rule out these kinds of necessary trade-offs; indeed, this is part of the strategic purpose of calling something a right. Perhaps we could circumscribe the right to avoid potential conflicts (eg, patients have the right to a rapid clinical review, but only under certain conditions), but in doing so we largely mitigate the purpose of framing it as a right in the first place.

In addition to thinking about the obligations of the health system to meet the needs of individual patients, we must also consider the needs of the community. We often think of the obligations of an HCP as being to the patient in front of them, but at a system level there is an obligation to consider the needs of the population of current and future patients. HCPs and National Health Service (NHS) policy-makers thus have to balance their obligations to individuals and to communities.^6^ This balancing act is incredibly difficult—effective and ethical solutions will be context-dependent and will require decision-makers to use their expert judgement. The introduction of a right to a second opinion threatens to tip this balance in favour of individuals, and to constrain the judgement of HCPs and decision-makers.

Conversely, if we understand the problem in terms of obligations rather than rights, we can more easily determine exactly what the purpose of the obligations are, what they require and how they are most effectively fulfilled in the broader context of other obligations. We would thus be in a better position to address the fundamental purposes of Martha’s Rule: protecting and listening to patients. If we focus too much on the patient’s right, we risk creating a system which may formally respect this right, but without effectively dealing with the underlying systemic issues. Focusing on obligations will also give systems the flexibility to take into account other concerns and obligations, and adapt processes for rapid clinical review in light of new and evolving evidence, and local needs and priorities (eg, local demographics causing language barriers or socioeconomic barriers to health literacy).

Moreover, framing access to a rapid clinical review as a right arguably places the cart before the horse. Why should we think that insisting on a right to a particular clinical service thereby obligates some party to provide that service? If we think that providing a particular clinical service is something that ought to be done—because it will promote patient safety, improve patient satisfaction or simply because it is the right thing to do—we might take the health system to have an obligation to provide it.

### Addressing power dynamics and poor culture

Understanding Martha’s Rule as an individual right of patients is not particularly helpful in ensuring the underlying aims of the rule are met. Focusing narrowly on the individual rights of the patient fails to address the systemic issues that contributed to Martha’s death, including the hierarchy within and between care teams that resulted in certain information being ignored, or additional support not being sought out; a lack of communication between departments; failure to take individual responsibility for patient care; a culture of privilege and infallibility among some consultants; and a condescension to or dismissal of (or even contempt for) patients. In the same way that we should be careful of reducing these problems to a matter of patient rights, we should also be wary of reducing these problems to a matter of patient empowerment—one that can be addressed by removing certain capacities from HCPs and placing it in the hands of patients.

Talk of ‘power imbalances’ (and consequent suggestions of empowerment) runs the very real risk of covering over a range of inequalities that are unavoidable, necessary and ultimately just in a medical context. HCPs have knowledge, clinical expertise, clinical experience and access to potential treatments that patients lack. Conversely, patients are sick and dependent on the HCPs’ exercise of their knowledge and expertise. Similarly, within the healthcare system itself, inequalities clearly exist between HCPs, based on specialty, professional role and experience. However, these inequalities should not be understood as unjust. They are not the result of the unfair distribution of resources nor of broader social injustices, but of acceptable choices made in pursuit of collective goals.

Thus, not all power imbalances are automatically and uniformly problematic. Navigating these inequalities in a way that is mutually beneficial for the patient and the HCPs requires care, trust and teamwork. It is when the patient–provider relationship breaks down that these inequalities can become unjust. Simply ‘empowering’ a patient by conferring on them a specific capacity (eg, to rapidly obtain a second clinical opinion) may serve to formally relocate power (and responsibility) in certain respects, but it fails to do justice to the complexity of these relationships by treating the matter as a simple balancing exercise, as moving the pendulum slightly in one direction rather than another.

The justification for this sort of service, then, is not that it empowers patients, and thereby helps to rectify the kinds of power imbalances that can lead to breakdowns of communication and respect between HCPs and patients (and between clinical teams). At best, it provides a way of circumventing these breakdowns once they have occurred. Focusing too narrowly on the issue of patient empowerment risks shaping the development of this service in such a way that it fails to be as beneficial to patients as it otherwise might be.

### Trust (with)in the health system

Martha Mills’ case raises a number of questions about trust within the health system. On the face of it, requesting a second opinion exemplifies a lack of trust, and perhaps even distrust, in the clinical team. Requesting a second opinion might also reflect a loss of confidence in the larger systems of communication, decision-making and oversight that structure clinical interactions. When trust in the clinical team has been lost and cannot be restored, or there is a loss of confidence in the relevant systems, an alternative means of interaction is required. By providing access to an independent clinical team, Martha’s Rule seeks to ensure that such an alternative is available. In doing so, it provides a mechanism for circumventing a relationship of distrust between patients and HCPs, and attempts to make the larger system more reliable in effectively treating patients. In effect, Martha’s Rule replaces trust in the clinical team with reliance on the health system. However, if we hope to restore trust between the patient and the clinical team, we need to do more than provide an option for avoiding distrust. HCPs need to demonstrate trustworthiness, which requires engaging in continuous self-monitoring about their own competences, so that they know them and their limits.^7^

At the same time, breakdowns in trust between the HCP and the patient are not necessarily due to the untrustworthiness of HCPs. Trustworthy HCPs may nevertheless be distrusted by patients, for both good and bad reasons. This mistaken distrust can be demotivating for HCPs and put further strain on the HCP–patient relationship. There is much more to say about the obligations of the trustworthy to those that distrust them, as well as the importance of avoiding mistaken distrust, than we can consider here. Nevertheless, framing access to a rapid second opinion as a ‘right’ risks glossing over the challenges introduced by the possibility of a patient’s request being based on unjustified distrust of the clinical team.

A distinct issue is how the provision of the kind of service described by Martha’s Rule affects public perceptions of the trustworthiness of the health system more broadly. Measures like Martha’s Rule are intuitively appealing insofar as they provide an assurance of performance at an institutional level. If a patient believes they are not receiving adequate care from one clinical team, there is an assurance in place that they can seek the opinion of another team. However, as we have argued elsewhere,^8^ assurances of performance diminish the need for trust. Because trust is a response to uncertainty, removing the uncertainty (eg, through an assurance or guarantee of performance) removes the need for trust. While implementing assurances of performance may serve to make behaviour more predictable, it does not make an individual or institution more trustworthy. It simply removes one task or domain about which they need to be trusted. In fact, in situations in which we require assurances of performance, we may be better off to demand that an institution or system be reliable or confidence-worthy, rather than trustworthy.

That being said, we might distinguish between assurances that function as replacements for trust and those that provide evidence of trustworthiness (ie, reasons for trusting). Suppose I am a trustworthy person, but can see that you are reluctant to trust me in some domain. To demonstrate to you that I am appropriately moved by your dependence on me, and so can be trusted, I might voluntarily constrain my ability to act in certain ways within that domain, because I can see that this is what you need in order to trust.

A generous interpretation of Martha’s Rule, then, is that it is an attempt by the NHS to demonstrate its general trustworthiness by providing assurances of performance in certain domains of activity. By recognising the limits of its doctors’ competence to provide care (ie, by recognising that in some cases mistakes are made), or defects in the way its systems function, the NHS as an institution is demonstrating that it can be trusted to do what is necessary to ensure that patients get the care they need. However, for Martha’s Rule to be a genuine demonstration of trustworthiness, and not merely an attempt to mollify the public, this service needs to be viewed within a wider context of how the health system understands and meets its obligations and commitments to patients. If we want this sort of service to help build trust in the health system, we need to think carefully about the ways in which we implement it. Characterising this as a ‘right’ risks undermining this effort.

### Implementation

We have argued to this point that Martha’s Rule (ie, a mechanism to obtain a rapid clinical review) should neither be conceived of as a right nor as a means of patient empowerment. Rather, in order to serve the dual goals of protecting patient safety and giving patients a voice regarding their own care—goals we unreservedly support—we should think of Martha’s Rule as highlighting particular obligations of the health system, but be open to different ways of meeting them.^2^ And, as we discussed above, the introduction of any new clinical service will necessarily affect the ways in which the health system is able to realise its existing obligations to individuals and communities.

While the UK government seems to have committed to implementing Martha’s Rule, the specifics of this have not yet been provided. There already exist various models for PARR services, which are currently used both in the UK and internationally. These services represent a practical, reasonably efficient and already available mechanism that could—with appropriate modifications—serve as a tool to identify and ensure that the obligations of the health system to patients are met.^3^

In the following, we outline the ethical considerations relevant to the widespread implementation of PARR services in the UK.

#### Accessibility

Implementing PARR systems widely in the NHS presents a significant challenge, primarily concerning accessibility. This encompasses several difficulties, including the need for effective communication to engage patients, the availability of adequate staffing, proper utilisation of these systems,^4^ and addressing patient concerns about undermining clinical staff or creating conflicts.^9^

While potential solutions exist to alleviate some of these accessibility challenges, others appear more complex. Research suggests that routine messaging, user-centric educational materials and clinician-led patient advocacy can help overcome activation barriers and enhance the effectiveness of rapid response systems.^10^ However, geographical disparities in healthcare accessibility pose a more complex challenge, further raising issues of consistency and fairness. For instance, in community hospitals, there may be a higher likelihood of staffing shortages and limited resources.

Furthermore, while certain measures can enhance referral calls for second opinions, they may not fully address the broader inequalities and discriminatory practices entrenched in the healthcare system. For instance, a University of Oxford study^11^ uncovered a fourfold higher mortality rate for black women in obstetric settings compared with white women. The study also highlighted persistent disparities in the reception of patient complaints based on race, with some black women’s pain complaints being ignored or dismissed as over-reactions. The implementation of PARR systems not only falls short in resolving these systemic concerns but also potentially raises further questions about patient complaint handling. These ways in which current PARR systems fail to adequately address these accessibility issues clearly need to be a core concern.

#### Flexibility in HCP response

While evaluations of existing PARR services are limited, evidence suggests that PARR calls are made for both safety and non-safety concerns, and HCPs require flexibility to respond appropriately. Patient concerns raised through PARR deserve to be heard, and require an appropriate and timely response, but not all concerns raised will require an urgent second opinion. For example, an evaluation of Condition Help in the USA found that the majority of calls were not safety-related, with almost half of all calls relating to pain management.^12^ Similarly, an evaluation of Ryan’s Rule in Australia reported that less than half of PARR calls resulted in a change in clinical care, and that for a majority of calls, communication was all that was required to resolve the issue.^13^ Patients and carers should be provided with clear information on the role of the PARR service, but they should not be responsible for triaging their own concerns as being safety or non-safety-related; this requires the clinical expertise of HCPs. For example, an increase in pain could indicate a safety issue through the worsening of an acute illness, or a non-safety issue where the patient’s condition is stable but requires improved pain relief.

Disagreement between the primary clinical team, and the recommendations of the PARR service, does not necessitate a change in clinical care. Genuine differences of medical opinion are accepted by UK law: provided a treatment recommendation is endorsed by a responsible body of experts, disagreement by other experts is not sufficient to make such a recommendation ‘unreasonable’.^14^ One possible consequence of making PARR services more accessible, however, is an increase in instances of such ‘reasonable disagreement’. While this possibility is not a decisive reason to prevent patients from seeking out second opinions, and thus from making PARR services available, navigating cases of expert disagreement may pose various practical challenges, although ones that are not unique to the PARR context, or even clinical practice (eg, what counts as ‘good evidence’, who assumes responsibility for the decision).^15 16^

#### Effectiveness and efficiency

Introducing a new clinical service comes with costs, both financial and administrative. Thus, an important part of implementing a PARR service across the NHS, and amending these services as necessary, will be establishing metrics for understanding the effectiveness of these services and measuring their value and impact for quality improvement. Again, this speaks to the importance of clearly delineating the purpose of this service (eg, promoting patient safety, providing patients a greater voice in their care).

In other related cases, a systematic review of family-initiated escalation in hospitals showed that the most common call reasons were ‘communication breakdown or concern about the patient’s plan of care, delays, medication or pain management concerns’.^17 18^ However, concerns about misuse of services and nuisance calls seem not to have been realised, and initial impact on critical care outreach staff workload seems to have been minimal.^3 19^ It is unclear whether this would change with the implementation of a ‘right’ to, and broader awareness of, these services. In the minority of cases where safety concerns were raised, PARR had an important role in the early identification of patient deterioration. A brief evaluation of Call 4 Concern found that of the 12 PARR calls assessed, 2 calls provided information that identified safety issues requiring urgent response to prevent further patient deterioration.^19^

PARR services may also provide a pathway for patient and carer voices to be heard within the health system. If successfully implemented, this service could have broader impact on quality of care through improving communication and strengthening trust between patients and HCPs, supporting a positive healthcare culture.^20^ How the service is presented—to both patients and HCPs—will be key to its success. If the implementation of Martha’s Rule was framed as a welcome invitation for the concerns of patients and families to be heard, and for them to provide valuable information that may reduce errors and improve patient care, this could serve the best interest of patients, HCPs and the health system more broadly.^9^ But if Martha’s Rule is framed as a right to a second opinion, its implementation may create an adversarial dynamic between patients and the health system. Patient expectations could develop that invoking Martha’s Rule is an entitlement in all circumstances, and the ability of HCPs to triage PARR concerns and respond appropriately will be constrained, without a corresponding increase in patient safety or strengthening of trust between patients and HCPs. Accordingly, it is important that Martha’s Rule, as a formal mechanism for patients to have their voices heard, does not become the only way patients can raise concerns and be taken seriously.

While the existing evidence suggests that the availability of PARR services has minimal effect on patient management, they may still be valuable in potentially identifying sentinel events requiring urgent escalation of clinical care. Or, even if PARR systems have a negligible impact on patient safety, they may nevertheless be an acceptable use of healthcare resources if they facilitate patients having a greater voice in their care.^21^ However we understand the benefits of PARR services, we must ensure they effectively and efficiently deliver these benefits.

## Conclusion

The circumstances of Martha Mills’ case illustrate the depth and complexity of the issues preventing the NHS from functioning as it should when it comes to communication between patients and HCPs. These issues and the broad systemic ones have been highlighted in a range of contexts already, perhaps most notably in the report by Robert Francis in the Mid-Staffordshire.^22^ However, implementing a ‘right’ to obtain a rapid clinical review does not address these issues. Framing Martha’s Rule as a right is practically and conceptually fraught, and fails to appreciate the need to think carefully about how introducing a PARR service fits into the wider health system. If we want to implement a service that is useful and effective—that protects patients and provides a way for their voices to be heard—we need to go beyond talk of rights.

## Footnotes

The NHS constitution does not confer a legal right to a second opinion. Similarly, the General Medical Council, as part of its good practice recommendations, states that doctors should ‘respect the patient’s right to *seek* a second opinion’ [(emphasis added]).

It may not be the case that these dual obligations are best realised via the implementation of a single rule or policy change: measures that might best promote patient safety could have the effect of reducing the impact of patient voices, while measures that give patients a greater voice in their own care may turn out to reduce patient safety.

Once this obligation has been established, patients might reasonably say that they have a ‘right’ to expect PARR services to be available, and efficiently and fairly run. But talk of rights at this point only makes sense because it is grounded in a sophisticated understanding of the obligations of the health system.

These services might also be leveraged to promote reflective practice (eg, reviewing reasons for PARR calls for quality improvement). Data quality, and the administrative burden on clinicians, will be important considerations here.

## References

[1] Roberts M (2023). NHS to introduce Martha’s rule for hospital patients. https://www.bbc.co.uk/news/health-66807426.

[2] Davies C (2023). Government backs Martha’s rule on right to second medical opinion in England. https://www.theguardian.com/society/2023/sep/14/government-backs-marthas-rule-on-second-medical-opinion-in-england.

[3] Curtis P (2023). Martha’s rule - explained. https://demos.co.uk/blogs/marthas-rule-explained/.

[4] Dyer C (2023). Martha’s rule: what could the proposed changes mean for doctors. BMJ.

[5] Curtis P, Wood C (2023). Martha’s rule: A new policy to amplify the patient voice and improve safety in hospitals. https://demos.co.uk/wp-content/uploads/2023/08/Marthas-Rule_finalversion.pdf.

[6] Newdick C, Sheehan M, Dunn M (2020). Tragic choices in intensive care during the COVID-19 pandemic: on fairness, consistency and community. J Med Ethics.

[7] Jones K (2012). Trustworthiness. Ethics.

[8] Graham M, Milne R, Fitzsimmons P (2023). Trust and the Goldacre review: why trusted research environments are not about trust. J Med Ethics.

[9] Ngoya Ntumba M, Edwards E, Haegdorens F (2023). Patient activated rapid response – the ‘999’ for patients admitted to hospital. Journal of Patient Safety and Risk Management.

[10] Yu S, Thornton K, King L (2022). Consumers’ views on reporting of patient deterioration before the development of a consumer-activated response service. Collegian.

[11] Knight M, Bunch K, Tuffnell D (2021). Saving lives, improving mothers’ care: lessons learned to inform maternity care from the UK and Ireland confidential enquiries into maternal deaths and morbidity 2017-19. https://www.npeu.ox.ac.uk/assets/downloads/mbrrace-uk/reports/maternal-report-2021/MBRRACE-UK_Maternal_Report_2021_-_FINAL_-_WEB_VERSION.pdf.

[12] Eden EL, Rack LL, Chen L-W (2017). Condition help: A Patient- and family-initiated rapid response system. J Hosp Med.

[13] Dwyer TA, Flenady T, Kahl J (2020). Evaluation of a patient and family activated escalation system: Ryan's rule. Aust Crit Care.

[15] Walker MJ, Rogers WA (2017). Reasonableness, credibility, and clinical disagreement. AMA J Ethics.

[16] Solomon M (2014). Parnas J.

[17] Gill FJ, Leslie GD, Marshall AP (2016). The impact of implementation of family-initiated escalation of care for the deteriorating patient in hospital: a systematic review. Worldviews Evid Based Nurs.

[18] Gill FJ, Leslie GD, Marshall AP (2016). ‘Family initiated escalation of care for the deteriorating patient in hospital: family centred care or just‘box ticking’’. Aust Crit Care.

[19] Odell M, Gerber K, Gager M (2010). Call 4 concern: patient and relative activated critical care outreach. Br J Nurs.

[20] Subbe CP, Ahsan S, Smith L (2021). An audible patient voice: how can we ensure that patients are treated as partners in their own safety?. Future Healthc J.

[21] Cooksley T, Astbury S, Holland M (2023). Martha's rule and patients' rights to a second opinion. BMJ.

[22] Mid Staffordshire NHS Foundation Trust Public Inquiry (2013). Report of the mid Staffordshire NHS foundation trust public inquiry: executive summary. https://assets.publishing.service.gov.uk/government/uploads/system/uploads/attachment_data/file/279124/0947.pdf.

